# Acute myeloid leukemia targets for bispecific antibodies

**DOI:** 10.1038/bcj.2017.2

**Published:** 2017-02-03

**Authors:** S S Hoseini, N K Cheung

**Affiliations:** 1 Department of Pediatrics, Memorial Sloan Kettering Cancer Center, New York, NY, USA

## Abstract

Despite substantial gains in our understanding of the genomics of acute myelogenous leukemia (AML), patient survival remains unsatisfactory especially among the older age group. T cell-based therapy of lymphoblastic leukemia is rapidly advancing; however, its application in AML is still lagging behind. Bispecific antibodies can redirect polyclonal effector cells to engage chosen targets on leukemia blasts. When the effector cells are natural-killer cells, both antibody-dependent and antibody-independent mechanisms could be exploited. When the effectors are T cells, direct tumor cytotoxicity can be engaged followed by a potential vaccination effect. In this review, we summarize the AML-associated tumor targets and the bispecific antibodies that have been studied. The potentials and limitations of each of these systems will be discussed.

## Introduction

Acute myeloid leukemia (AML), characterized by the infiltration of bone marrow, blood, and other tissues by malignant cells of the myeloid lineage is the most common acute hematologic malignancy of adults. In patients diagnosed before 60 years of age, AML is curable in 35–40% of cases, whereas only 5–15% of those presenting later in life can be cured.^1^ The treatment of AML that has remained essentially unchanged over the last three decades consists of intensive induction therapy followed by hematopoietic stem cell transplantation (HSCT). Many novel therapeutic agents, both small molecules targeting signaling pathways and immunologics are actively being investigated as salvage therapies or as alternatives to the standard of care. One class of immunotherapeutic agents is that of bispecific antibodies.

**Figure 1:**
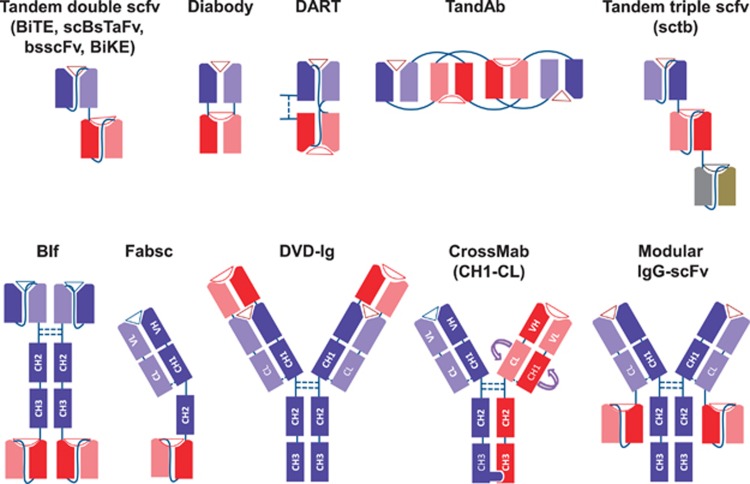
Different bispecific antibody formats. Heavy chain sequences are depicted in dark blue, dark red and dark gray, whereas corresponding light chains are in similar but lighter colors. Linkers are shown by continuous lines and disulfide bonds, when shown, are illustrated in dotted lines. Antigen epitopes are shown by triangles and semicircles. scFv, single-chain Fv fragments; scBsTaFv, single-chain bispecific tandem fragment variable; BiTE, Bispecific T-cell Engager; bsscFv, bispecific single-chain Fv; BiKE, bispecific killer-cell engager; DART, Dual-Affinity Re-Targeting; TandAb, Tandem Diabodies; sctb, Single-chain Fv Triplebody; BIf, Bispecific scFv Immunofusion; DVD-Ig, Dual-Variable-Domain Immunoglobulin; VH, variable heavy chain; VL, variable light chain; CL, constant light chain; CH1-3, constant heavy chains 1 to 3.

Bispecific antibodies combine the binding specificities and biologic functions of two antibodies into one molecule, one for a tumor-associated surface antigen, and the other for a surface antigen on the effector cells, such as T cells or natural-killer (NK) cells. Through the dual specificities, tumor cells are brought into close proximity to the effectors. Most importantly, if binding to the second specificity is agonistic, the cytotoxic functions of effectors can be activated at close proximity to the leukemic cells. Various combinations of whole antibodies and their fragments have yielded more than 60 different formats of such AML bispecific antibodies (examples in Figure 1).^2^ The immunologic properties and clinical potentials of each of these AML-associated targets are summarized in Table 1. Besides, a list of clinical trials investigating bispecific antibodies in myeloid leukemia is mentioned in Table 2. Characteristics of the bispecific antibodies (molecular weight, affinity, EC_50_ and parental clone) are summarized in Table 3.

In this review, we summarize those AML targets for which bispecific antibodies have been developed, in descending order of clinical relevance: CD33, CD123, Wilms' tumor protein (WT1), CD13, CD15, CD30, CD45, CD47, C-type lectin-like molecule 1 (CLL1), Fms-like tyrosine kinase 3 (FLT-3) and angiogenic growth factors.

## CD33

CD33 or Siglec-3, a 67-kDa glycoprotein, is a member of the sialic acid-binding immunoglobulin-like lectin (Siglec) family, which in turn belongs to the immunoglobulin superfamily. The extracellular domain of CD33 comprises one amino-terminal sialic acid-binding V-type and one C2-type immunoglobulin-like domain connected by a helical transmembrane sequence to a cytoplasmic tail containing two immunoreceptor tyrosine-based inhibitor motifs (ITIM). Phosphorylation of the tyrosine residues by the Src family kinases is involved in the recruitment and activation of the Src homology-2 (SH2) domain-containing tyrosine phosphatases SHP1 and SHP2.^3^

CD33 is a receptor that appears during commitment of the hematopoietic stem cell to the myelomonocytic lineage. It is expressed on myeloid progenitors, monocytes, myeloid dendritic cells and less so, on macrophages and granulocytes.^3^ Although it is restricted mainly to the myeloid lineage, low levels of CD33 expression has been reported on some lymphoid cells, including the earliest precursors of human fetal thymocytes and human CD34(+) postnatal thymocytes.^4^ In addition, activated NK cells and T cells can also express CD33.^5^ CD33 is expressed on the majority of AML cells and the level of CD33 seems to correlate with the disease prognosis.^6^

The first bispecific T-cell engager (BiTE) developed against CD33 is AMG 330 (Amgen Inc., Thousand Oaks, CA, USA), which binds to a linear epitope containing the amino acid sequence IPYYDKN in the CD33 V-type domain.^7, 8, 9^ This BiTE activates and expands T cells from autologous clinical samples of patients with AML and mediates *in vitro* lysis of primary AML and normal myeloid cells in a dose dependent manner at concentrations as low as 1 ng/ml (EC_50_=0.35–2.7 pm).^8, 10^ However, it is noteworthy that activated T and NK cells can also express CD33.^5^ CD33-independent activation with an anti-CD19 BiTE led to CD33 neo-expression on a minor subset (<6%) of T cells, associated with their fratricide but with minimal effect on AML-directed cytotoxic T-cell function.^9^ Importantly, soluble CD33 found in the blood of some patients with AML does not affect the AMG 330-mediated T-cell activation or cytotoxicity.^9^ Antibody-mediated endocytosis of antigens could reduce the availability of cell surface targets for antibody therapy. In contrast to bivalent anti-CD33 IgG antibodies, AMG 330 is not endocytosed and does not modulate surface expression of CD33. In addition, the function of this BiTE is neither affected by common CD33 single nucleotide polymorphisms nor by the adenosine triphosphate-binding cassette transporter activity. *In vivo* experiments showed that AMG 330, in the presence of activated human T cells, could suppress the growth of a subcutaneous AML cell line xenograft in humanized mice improving survival.^8, 9^

To better understand the immunobiology of AMG 330 BiTE, human leukemic cell lines were engineered to express the inhibitory (PD-L1 and PD-L2) or activating (CD80 and CD86) ligands to interact with their respective receptors (that is, CD28 and PD-1), It was found that the expression of PD-L1 and PD-L2 inhibitory molecules on target cells reduced the *in vitro* cytotoxicity of AMG 330 BiTE in the presence of healthy donor T cells at low effector:target (E:T) ratios. This inhibition correlated with the level of ligand expression on target cells.^11^ On the other hand, CD80 and CD86 activating molecules expressed on tumor cells increased the cytolytic activity of AMG 330. Treatment with anti-CD28 activating antibodies enhanced the cytotoxicity of AMG 330 against human-AML cell lines and primary AML samples from patients with refractory leukemia.^11^

In a subset of patients, T-cell activation and cytotoxicity were suboptimal,^6, 8, 10^ possibly because of inhibition by the PD-1/PD-L1 pathway. Although the majority of primary AML cells do not express PD-L1, it can be upregulated by the proinflammatory cytokines released during the activation of T-cells by AMG 330. The addition of anti-PD-1/PD-L1 antibodies restored T-cell expansion, their interferon-γ (IFNγ) secretion and the lysis of leukemic cells. Importantly, whereas low E:T ratios and low CD33 expression on targets impeded T-cell cytotoxicity, extending the *in vitro* incubation time substantially improved tumor killing.^10, 12^ In addition, the use of epigenetic modifiers such as panobinostat and azacitidine could increase the expression of CD33 to enhance AMG 330-induced cytotoxicity.^10^ These observations are clinically relevant since leukemic stem cells express lower amounts of CD33 when compared with the bulk of the leukemia population.^6^ These observations have prompted the use of prolonged administration schedule of AMG 330 to improve its anti-leukemic effect. However, as CD33 is present on normal hematopoietic stem cells (although lower than that on leukemic stem cells^6^), such an infusion schedule could prolong also cytopenia.^13^ A recent study showed that the effectiveness of AMG 330 was inferior against AML samples from patients with relapsed/refractory leukemia when compared with samples taken from patients at the time of first diagnosis. These data suggest the existence of possible resistance mechanisms among AML blasts.^14^

A second bispecific antibody format against CD3 and CD33 attaches the VH of an anti-CD3 to the VL of an anti-CD33 antibody, whereas connecting the VH of the anti-CD33 to the VL of the anti-CD3 antibody, with the two tandem single-chain Fv fragments (scFv) joined by a glycine-serine (G4S)_5_ linker. The resulting construct was initially complicated by the formation of fragmented nonfunctional proteins because of DNA recombination triggered by sequence homology. This problem was resolved by rearranging the variable regions in a way such that the variable heavy (VH) of CD33 and VH of CD3 were linked to their cognate variable light (VL) regions. In addition, the (G4S)_5_ linker was replaced by a new helical sequence (amino acid sequence: EKEALKKIIEDQQESLNK) in joining the CD33 and CD3 binding moieties. The newly generated CD33 × CD3 single-chain bispecific tandem fragment variable (scBsTaFv) construct was able to lyse CD33-expressing target cells in the presence of peripheral blood mononuclear cells (PBMC) obtained from healthy donors.^15, 16^ When a green fluorescent protein was incorporated into the bispecific antibody, the formation of immunological synapse between T cells and target cells was visualized.^17^ When the CD33-CD3 scBsTaFv bispecific antibody was further humanized, it retained its ability to arm T cells to kill AML blasts at picomolar concentrations (EC_50_=15 pm).^18, 19^ When tested by *in vitro* colony forming assays, the multilineage reconstitution potential of hematopoietic stem cells treated with the humanized antibody was not compromised.^18^ To further improve the potency of this bispecific antibody, 4-1BB ligand (4-1BBL) co-stimulatory molecule was incorporated into the construct in a modular manner. The novel modular system outperformed the original humanized CD33 × CD3 scBsTaFv in killing CD33^low^ AML cells at low E:T ratios *in vitro*.^19^

One obstacle in the use of these small-size bispecific antibody fragments is their rapid clearance through kidney, necessitating their continuous infusion for up to several weeks.^20^ To tackle this problem, mesenchymal stromal cells (MSC) were engineered to continuously secrete the humanized CD33-CD3 scBsTaFv, and to express 4-1BBL for T-cell co-stimulation. T cells, AML cells and scBsTaFv-engineered MSC cells were co-cultured for 24 h before injection into immunodeficient mice. Results showed that antibody-engineered MSC cells could redirect T cells toward AML cells and extend the survival of leukemic mice.^21^ Further experiments will be necessary to assess whether this therapeutic modality can target neoplastic cells in established leukemia animal models, and to determine the optimal delivery method for the engineered MSCs, as well as to investigate if MSCs can home to tumor sites and activate T cells without inducing immunosuppressive effects.

Recently, a panel of bispecific tandem diabodies (TandAb) specific for CD3 and CD33 have been generated of which one (AMV-564) was chosen for further clinical development. The AML lysis mediated by this construct in the presence of T cells depended on antibody dose and E:T ratio. Furthermore, the TandAb-induced cytotoxicity was similar against AML samples from newly-diagnosed patients, or from those with refractory/relapsed leukemia. No correlation was found between the antibody-mediated cytotoxicity and CD33 expression level on target cells. This TandAb was also successful against AML subcutaneous xenografts in mice.^22^

Moving beyond CD3 on T cells, other activating ligands or antibodies designed for lymphocytes have been tested against human-AML. Since both NK and CD8(+)T cells express the NKG2D activating receptor, a dual specific scFv-ligand was designed where the NKG2D activating ligand, UL16 binding protein 2 (ULBP2), was fused to the anti-CD33 scFv via an 18 amino acid (EKEALKKIIEDQQESLNK) linker. The ULB-CD33 scFv construct redirected the cytotoxicity of pre-activated NK cells and PBMCs toward AML targets. Although administration of this scFv-ligand to freshly-isolated CD8(+)cells in the presence of AML blasts enhanced IFNγ secretion, no killing of leukemic cells was observed. Furthermore, although co-administration of ULB-CD33 scFv and CD33 × CD3 scBsTaFv augmented the IFNγ and tumor necrosis factor-α (TNFα) secretion by CD8(+)T cells, the anti-leukemic cytotoxicity was not superior when compared with CD33 × CD3 scBsTaFv alone. Co-stimulation through the NKG2D receptor could reinforce the CD3-mediated signaling on T cells, but it was not enough to increase the lysing of target cells.^16^

Although many efforts have focused on bispecific antibodies to retarget T cells in cancer immunotherapy, the engaging of the innate immune system (that is, NK cells through the CD16 Fc-receptor) has also shown promise. A chemical conjugate of anti-CD16 and anti-CD33 monoclonal antibodies was able to redirect cytotoxicity of resting or activated NK cells, as well as peripheral blood lymphocytes toward AML leukemic blasts, with maximum cytotoxicity requiring 10 ng/ml concentrations of antibody.^23^ Nevertheless, chemical conjugates generally yield a heterogeneous mixture of cross-linked products, of which only a subset has the desired target specificity. Using recombinant DNA technology, large scale and efficient production of bispecific antibodies has since taken over.^24^ A fully-humanized scFv of CD16 and CD33 were joined together using a 20-amino acid linker derived from the human muscle aldolase. This BiKE could activate NK cells to secret IFNγ and TNFα, and to lyse CD33(+) AML targets. Treatment of NK cells with an ADAM17 inhibitor and CD16 × CD33 BiKE prevented CD16 shedding from NK-cell surface, thereby enhancing NK-cell cytokine secretion and cytotoxicity.^25^ This BiKE was further tested using NK-cell samples from patients with myelodysplastic syndrome (MDS) where the NK-cell frequency and their CD16 expression were low. Upon treatment with the CD16 × CD33 BiKE, NK cells from these patients produced IFNγ and TNFα, and lysed a CD33(+) AML tumor cell line as well as CD33(+) myeloid derived suppressor cells. The latter cells possess NK and T-cell suppressing properties, and are significantly increased in patients with MDS.^26^

A further improvement of BiKE attempted to improve *in vivo* expansion of NK cells. Instead of using interleukin-2 (IL2), which induces regulatory T cells (Tregs) that suppress NK-cell function,^27^ interleukin-15 (IL15) can be used to enhance NK-cell homeostasis in a trispecific killer-cell engager (TriKE). Here, the human IL15 sequence, flanked by two linkers, was inserted between the scFvs of the CD16 × CD33 BiKE.^28^ The IL15 TriKE induced similar NK-cell mediated killing when compared with a mixture of IL15 and BiKE. In a murine AML xenograft model using human NK cells, TriKE was superior to BiKE.^28^

Instead of the IL15 sequence, a third scFv can be inserted to create a triple scFv antibody. Two anti-CD33 scFv fragments attached in tandem via a flexible 20-amino acid linker (Gly_4_Ser)_4_ to a single anti-CD16 scFv (sctb: single-chain Fv triplebody) was compared with the single anti-CD33 scFv construct (bsscFv: bispecific single-chain Fv). CD33 avidity of the sctb was 3.5 fold higher when compared with that of the bsscFv, even though their affinity was comparable. Both antibody constructs in the presence of human PBMC mediated lysis of CD33(+) AML cell lines with EC_50_ values in picomolar ranges (212–426 pm for bsscFv, 1.8–18 pm for sctb). Importantly, the sctb was 10–200-fold more potent than the bsscFv in killing human leukemic cell lines.^29^ The logic of this enhancement was extended to targeting more than one tumor antigen on the surface of cancer cells. For acute leukemia with mixed-lineage phenotypes, a sctb with dual target specificities (anti-CD33 scFv and anti-CD19 scFv) was attached to an anti-CD16 scFv. Compared with the CD33 × CD16 or CD19 × CD16 bsscFv, the CD33 × CD16 × CD19 sctb redirected cytotoxicity of MNCs against a double-positive cell line at 23 and 1.4 fold lower concentrations, respectively.^30^ In another series of experiments three sctb antibodies were generated. Two of the sctb antibodies targeted a single antigen on leukemia cells (either CD33 or CD123), whereas the third sctb targeted both leukemia antigens. Although both single and double antigen formats were able to mediate lysis of AML targets in the presence of PBMC, the latter was more potent, showing the advantage of simultaneous targeting of more than one tumor antigen.^31^

CD64 (FcγRI), which is expressed on monocytes, macrophages and neutrophils, has an important role in antibody-dependent cell mediated cytotoxicity (ADCC). A bispecific antibody was generated by chemical conjugation of CD33 and CD64 and was able to redirect cytokine-activated monocyte cytotoxicity toward CD33 leukemic cells. IFNγ, granulocyte-colony-stimulating factor (G-CSF) and granulocyte-macrophage colony-stimulating factor (GM-CSF) increased the expression of CD64 on monocytes and enhanced their cytotoxic potential.^32^ Treatment of target cells with IFNγ also increased their killing by the bispecific antibody.^33^ Although the treatment of AML cells with monospecific anti-CD33 or anti-CD64 antibodies for 48 h inhibited AML cell growth, the inhibitory potency mediated by the bispecific molecule was higher. Furthermore, the bispecific antibody augmented the anti-tumor effects of monocytes in a murine tumor model.^34^

## CD123

CD123 is the alpha subunit of the interleukin-3 (IL-3) receptor. This 75 kDa single-pass type-I membrane protein contains three extracellular, one transmembrane and one intracellular domain. It is expressed on monocytes, B cells, megakaryocytes, plasmacytoid dendritic cells, and hematopoietic stem and progenitor cells.^35^ CD123 expression in B lymphoid and myeloid progenitors is high, whereas erythroid progenitors and primitive hematopoietic cells express either low or undetectable levels.^36^ Overexpression of CD123 can promote cell proliferation and may induce leukemogenesis.^37^ CD123 was once thought to be an AML stem cell marker.^38^ AML cells with high CD123 expression had higher proliferation and were more resistant to apoptosis induced by growth factor deprivation. Higher expression level of CD123 among AML blasts is associated with lower complete remission rates and poorer overall survival.^39^

To redirect T cells toward CD123 leukemic cells, the scFv of an anti-CD123 antibody (CD123scFv) was fused to the N terminus of the truncated human IgG1-Fc (CH2-CH3) connected in turn to an anti-CD3 scFv (CD3 scFv) at its C terminus, a platform named bispecific scFv immunofusion (BIf). The C-terminal location of the CD3 scFv reduced its affinity for CD3(+) T cells by two orders of magnitude. The BIf was able to mediate lysis of CD33(+) target cells by T cells at low picomolar EC_50_ (EC_50_=8 pm).^40^ Another bispecific tandem scFv construct was built comprising the scFv fragments of anti-CD123 and anti-CD16 antibodies. The bispecific construct at picomolar concentrations (EC_50_=211–364 pm) triggered the lysis of leukemic cells in the presence of PBMC.^41^ As mentioned earlier, a CD16 scFv-based sctb against CD123 demonstrated potent anti-leukemic activity, but inferior to a similar but double-targeting sctb simultaneously targeting CD33 and CD123.^31^

A dual-affinity re-targeting (DART) molecule named MGD006 (MacroGenics, Rockville, MD, USA), was built using two independent chains, one consisting of the humanized VL of anti-CD3 attached in tandem to the VH of anti-CD123, and the other consisting of the VL of anti-CD123 attached to the VH of anti-CD3 antibody. The two polypeptides were attached via disulfide bonds between the C terminus of the VH fragments. This DART molecule was able to activate and expand T cells and suppress AML xenografts in mice reconstituted with human PBMC. Because of the small size of the molecule (59 kD), continuous infusion over seven days was necessary. In cynomolgus macaques, MGD006 produced transient IL-6 release and reversible decrease in the red blood cell mass.^42^ In humanized NSG mice, MGD006 was effective against xenografts derived from a leukemic cell line or from primary AML samples.^43^ A first in human study is ongoing in AML patients (ClinicalTrials.gov identifier NCT02152956).

## WT1

WT1 is a zinc finger transcription factor that has important roles in cell survival and development. Several isoforms of WT1 exist, each with specific inhibitory or activating functions on a variety of important genes and cellular pathways. Mutations in the *WT1* gene have been associated with human diseases. In AML, *WT1* is considered an oncogene and its expression was found on the majority of AML samples.^44^ Importantly, higher *WT1* gene expression is associated with lower complete remission rates and decreased survival.^45^ Most therapeutic antibodies recognize intact antigens expressed on the cell surface; however, the majority of leukemia-associated antigens are intracellular. When degraded, their peptides are presented on human leukocyte antigens (HLA) as peptide–MHC (major histocompatibility complex) complexes on the cell surface. Recently, a BiTE construct comprised of the scFv fragments of antibodies specific for CD3 and WT1 epitope RMF in the context of HLA-A*02:01 (WT1-BiTE) was made.^46^ This BiTE could bind to WT1(+) and HLA-A*02:01(+)AML targets and activate T cells to proliferate and kill the leukemic cells *in vitro* and in immunodeficient mice. No binding to CD34(+)cells was found. Furthermore, WT1-BiTE therapy induced long-term T-cell responses against tumor-associated epitopes other than WT1 because of epitope spreading, which could greatly enhance the therapeutic efficacy of this bispecific construct. However, cross reactivity of these T-cell receptor (TCR)-like antibodies with other peptides on other HLA antigens could be a major hurdle for clinical development.^47^

## CD13

CD13, also known as aminopeptidase-N or gp150, is a myeloid membrane-bound zinc-dependent metalloprotease with an extracellular enzymatic moiety that cleaves N-terminal amino acid residues from oligopeptides.^48^ CD13 contains an extracellular domain joined via a helical transmembrane sequence to a small cytoplasmic domain. CD13 has several roles including tumor cell invasion, adhesion, differentiation, proliferation, apoptosis, motility, phagocytosis and angiogenesis.^49^ Monoclonal antibodies targeting CD13 result in apoptosis of primary AML cells and cell lines.^50^ In an attempt to target CD13 in AML, a bispecific antibody was generated by chemically conjugating the anti-CD3 and anti-CD13 Fab' fragments. This bispecific antibody markedly enhanced the cytotoxicity of IL2- or IL7-stimulated PBMCs against CD13(+) AML cells. Although the bispecific construct inhibited granulocyte-macrophage and mixed cell colonies of normal bone marrow progenitors, the inhibition was less than that for AML blast colonies.^51^

## CD15

CD15 (3 alpha-fucosyl-*N*-Acetyl lactosamine), also called Lewis X or stage-specific embryonic antigen 1 (SSEA-1), is a carbohydrate antigen expressed on the majority of AML cells and on some NK cells, T cells, monocytes, neutrophils and eosinophils.^52^ CD15 expression on AML blast was associated with favorable prognosis such as continuous complete remission and longer survival.^53^ In an attempt to design a bispecific antibody, the fragment of antigen-binding (Fab) of an anti-CD64 antibody was chemically conjugated with whole IgM monoclonal antibody against CD15. This bispecific construct was able to redirect *in vitro* cytotoxicity of IFNγ-activated monocytes toward CD15(+)AML cells in the presence of human serum as a source of complement. The antibody conjugate was assessed in a phase I clinical trial of four patients with CD15(+) cancer. Six doses of the drug over a period of two weeks were administered. In a patient with AML, a 30–60% transient reduction in peripheral blasts was observed when peak serum level of the antibody was as low as 50 ng/ml. No toxicity was reported.^54^

## CD30

CD30, member 8 (TNFRSF8) of the tumor necrosis factor receptor superfamily, is a 120-kDa type-I transmembrane glycoprotein. It is expressed on resting human CD8(+) T cells, activated B and T cells and their leukemias and lymphomas. CD30 has a co-stimulatory, mitogenic and activation effects on T cells.^55^ CD30 is also expressed with variable intensity on 36%^56^ to 50%^57^ of patient AML and its expression is associated with *FLT-3*-ITD mutations and leucocytosis.^57^ Upon cleavage from the extracellular domain, CD30 is released as a soluble form that can be detected in the blood of patients with Hodgkin and non-Hodgkin lymphoma and it is associated with poorer prognosis.^58, 59^ A TandAb containing two binding sites for CD30 and CD16A has been generated. Unlike an anti-CD30 IgG, the TandAb did not bind to the non-activating CD16B receptor. Furthermore, binding of the TandAb to CD30(+) target cells was higher than that of the anti-CD30 IgG, resulting in improved killing of target cells. Although not tested against AML blasts, this TandAb exhibited cytotoxicity against CD30(+) non-Hodgkin lymphoma cells. In the absence of CD30(+) target cells, TandAb did not activate NK cells,^60^ an important consideration for the clinical application of such bispecific antibodies.

## CD45

CD45 is a protein tyrosine phosphatase enzyme expressed in various isoforms. It contains an extracellular sequence connected via a helical transmembrane domain to a cytoplasmic tail. CD45 is expressed on all hematopoietic cells except mature red blood cells and platelets.^61^ A recombinant bispecific antibody was generated comprising a scFv against the radiometal complex with 1,4,7,10-tetraazacyclododecane-1,4,7,10-tetraacetic acid (DOTA, a metal chelating agent) and a CD45 binding moiety containing the light chain of an anti-CD45 antibody and the heavy chain of rituximab.^62^ *Invivo* targeting was performed in human-AML-bearing mice after injecting the antibody construct, followed by administering a clearing agent after 22 h and finally injecting the yttrium-90-labeled DOTA. Biodistribution studies revealed favorable antibody retention in tumor sites, whereas the normal organs showed minimum uptake. The therapeutic potential of this bispecific agent was not reported.

## CD47

CD47 is a 50-kDa glycoprotein belonging to the immunoglobulin superfamily. It contains an extracellular IgV domain next to a five-fold-passing transmembrane domain and a short cytoplasmic sequence.^63^ CD47 binding to signal regulatory protein-α expressed on dendritic cells and macrophages delivers a signal which inhibits phagocytosis.^64^ CD47 is a leukemia stem cell (LSC) marker and its overexpression in AML has been associated with poor survival.^65^ Recently, a bispecific antibody was generated to target CD47 and CD20 to treat lymphoma. Here, the C terminus of the VH and VL of an anti-CD20 antibody were joined by short peptide linkers to the N terminus of the respective chains in the anti-CD47 IgG to generate a bispecific dual-variable-domain immunoglobulin (DVD-Ig) format. Affinity of the DVD-Ig relative to the parental monoclonal antibody for CD47 was diminished 15–20-fold. In comparison to a 12-amino acid linker, DVD-Ig using the shorter 5-amino acid linker showed weaker binding to CD47. Fortunately this lower affinity allowed the 5-amino acid linker form to prefer the double-positive CD47(+)CD20(+)cells and avoid being trapped in a CD47 antigen sink. The bispecific short-linker construct improved the survival of lymphoma-bearing mice and recapitulated the therapeutic benefit of anti-CD47 and anti-CD20 antibody combination therapy.^66^

## CLL1

CLL1, also known as C-type lectin domain family 12 member A (CLEC12A), is a pan-myeloid antigen containing an extracellular, a transmembrane and a cytoplasmic domain. CLL1 is expressed on myeloid cells and peripheral blood monocytes, dendritic cells and granulocytes. Thanks to its absence on normal hematopoietic stem cells and its expression on LSC, CLL1 was explored as a marker for minimal residual disease in AML.^67^ To test the effect of targeting CLL1 in AML, the Fab fragments of anti-CCL1 and anti-CD3 antibodies were site-specifically modified using the unnatural amino acid, p-acetylphenylalanine and then conjugated to generate a bispecific Fab (BiFab). Compared with a BiFab against CD33 and CD3 generated using the same methodology, the CLL1-CD3 antibody-mediated stronger *in vitro* cytotoxicity and superior *in vivo* anti-tumor activity in immunodeficient mice with established leukemia xenografts receiving expanded T cells.^68^ Another laboratory also successfully demonstrated the *in vitro* functionality of a CLL1-CD3 BiTE in the presence of T cells against AML cells.^69^

## FLT-3

FLT-3, also known as CD135, is a receptor-type tyrosine kinase and has an important role in the development of hematopoietic progenitor cells and dendritic cells. FLT-3 contains an extracellular sequence with an Ig-like C2-type domain, a helical transmembrane part and a cytoplasmic domain with protein kinase activity. FLT-3 is expressed on committed lymphoid and myeloid progenitors and on monocytes.^70, 71^ Activating FLT-3 mutations are present in about 30% of patients with AML.^72^ Furthermore, the level of FLT-3 expression on AML cells is increased and is associated with higher percentage of bone marrow blasts and high leukocyte counts.^70, 73^ Two bispecific antibody formats have been designed to redirect T cells towards FLT-3(+)AML cells. One of the constructs, termed Fabsc, contained an anti-FLT-3 Fab fused to a scFv specific for CD3 via a CH2 linker modified to prevent dimerization and binding to Fc receptors. In the other construct, scFv fragments of the two antibodies were fused together using a BiTE format. Whereas both constructs were able to lyse FLT-3(+)target cells, the Fabsc format had higher affinity for FLT-3, lower aggregate formation and superior production yield.^71^

## Angiogenic growth factors

Neoangiogenesis has a crucial role in pathogenesis of cancers including AML. The expression of two angiogenic agents, vascular endothelial growth factor-A (VEGF-A) and the angiopoietin-2 (Ang-2) on AML cells is associated with poorer patient outcome.^74, 75^ Circulating levels of Ang-2 and VEGF are also strong predictors of poor survival in AML patients.^76, 77, 78^ The expression of VEGF-A and its receptors on AML cells is increased, raising the possibility of an autocrine proliferation mechanism in AML.^79^ Bispecific antibodies have been generated to simultaneously target VEGF-A and Ang-2. In one approach called CrossMab technology, a heterodimeric IgG1-based bispecific antibody with monovalent binding moieties for VEGF-A and Ang-2 antigens was constructed. In this platform, monomeric heavy chains of anti VEGF-A and Ang-2 antibodies are preferentially paired via the Knobs-into-holes methodology to make a heterodimeric heavy chain, whereas the correct pairing of light chains is guaranteed by crossover of the heavy chain constant-1 (CH1) and light chain constant (CL) domains.^80^ Although not yet tested against AML targets, this CrossMab antibody showed *in vivo* functionality against various cell lines including breast, lung, prostate, gastric, pancreatic and colon cancer, inhibited hematogenous spread of tumor cells, and was able to suppress corneal angiogenesis in mice.^80, 81^ Using another approach, so-called bispecific CovX-bodies were chemically synthesized by first linking the chemically synthesized VEGF and Ang-2 binding peptides by means of an azetidinone linker and then fusing these heterodimeric peptides to a scaffold antibody in a site-specific manner. *In vivo* functionality studies showed that the CovX-body imparted anti-angiogenic effects and reduced the growth of colon, breast and skin cancer xenografts.^82^

## Perspective and conclusions

T lymphocytes are highly efficient professional killers proven successful clinically in a variety of human cancer types. Although this recognized potential of T cells is just beginning to be exploited for immunotherapy of leukemia, NK cells offer unique opportunities in the AML setting, both in terms of its trafficking pattern and its functional competency. In contrast to solid cancers leukemia is generally a blood borne disease where NK and T cells have an easier access to the cancer cells. Since leukemic blasts are mainly present in the bone marrow and blood, effector cells have better access to cancer cells. Unlike T cells, NK cells do not penetrate all tissues.^83^ Hence, although T-cell-bispecific antibodies may be necessary for solid tumors, both NK and T-cell bispecifics could be used for blood born malignancies such as leukemia. The important role of NK cells and the killer-cell immunoglobulin-like receptor)–ligand interactions in preventing relapse following allogeneic transplantation for AML is well documented.^84^ As higher E:T ratio improves the function of bispecific antibodies,^10, 13, 85^ a robust endogenous effector population or the adoptive transfer of exogenous effector cells should increase the efficacy of these therapies. Application of allogeneic effector cells as an off-the-shelf immunotherapy has been extensively investigated after myeloablative preconditioning.^86^ With age, thymic function involutes and if T cells are further damaged by myeloablative conditioning,^87^ they need to be resuscitated with growth factors, such as interleukin-7, interleukin-15, keratinocyte growth factor or sex steroid hormone inhibition.^88^ Alternatively, the administration of allogeneic precursor T cells could improve T and NK-cell reconstitution without inducing graft-versus-host disease (GvHD) at least in mouse models;^89^ however, their potential for human application is still uncertain. Unlike T cells, NK-cell recovery does not require the thymus, and is relatively fast after dose-intensive or myeloablative chemotherapy.^90^ Unlike allogeneic T cells that induce GvHD, allogeneic NK cells do not generate and may even reduce GvHD.^91^ Hence, administrating such NK cells to enhance effector-target ratio should be clinically safe. Although CD3 is a proven activating receptor on T cells for bispecific antibodies, the clinical utility of agonistic receptors other than CD16 on NK cells is still investigational. NKG2D is one such activating receptor^92^ and MICA, MICB, ULBP1-6 are the other known activating ligands.^93, 94, 95^ Activating NK cells using anti-CD16 antibodies allows selective activation of FcRIII (CD16), unlike IgGs which stimulate all FcR receptors of the activating (CD16,CD32A, CD64) and inhibitory classes (for example, CD32B). In addition, activation of CD16 through specific anti-CD16 antibody bypasses the genetic polymorphism among patients with differential FcR affinity for IgG.^60, 96, 97^ With increasing sophistication in the genetic engineering of antibodies, multi-specific formats to include antibodies that will neutralize the inhibitory receptors may be possible, including inhibitory KIRs (2DL2/2DL3, 3DL1),^98, 99^ PD-1 (B7-H1),^100^ and B7-H3.^101^

Selecting the optimal leukemia-associated target antigen is as critical as choosing the proper class of effector cells or the particular antibody platform. Because of the importance of LSC in the recurrence of AML, bispecific antibody constructs need to engage antigens expressed on LSCs. Some of the potential LSC candidate antigens for generation of bispecific antibodies have been discussed by other investigators, including CD44,^102^ CD52,^103^ CD96,^104^ CD300f^105^ and TIM-3.^106^ A subset of leukemia called biphenotypic or mixed-lineage leukemia are composed of a heterogeneous mixtures of lymphoid and myeloid blasts. In addition, lineage switching could happen *de novo* in neonates, or over the natural course of the disease or following ralapse after treatment.^107^ Bispecific antibodies simultaneously targeting CD3 (on T cells) and a single marker shared by both lymphoid and myeloid stem cells (for eample, CD9^(refs^ ^108, 109^^)^) or trispecific antibodies targeting two separate markers on the leukemia blast (CD33 for myeloid and CD19 for lymphoid) could prevent escape of biphenotypic LSC. Furthermore, the carbohydrate antigen Lewis Y (CD174) has been introduced as a new marker for AML blasts and has been targeted with CARs in a clinical trial.^110^ Development of bispecific antibodies against this antigen in AML merits attention. CD33 may have a theoretical advantage over other myeloid markers for building bispecific antibodies since it carried on myeloid suppressor cells, an important population in the tumor microenvironment that impacts host T-cell immunity.^111^

Antigen density on leukemic cells can affect antibody binding and effectiveness of bispecific antibodies. When antigens are expressed at low levels, like WT1-peptide-MHC complexes, higher-affinity antibodies may be necessary for optimal cytotoxicity.^112^ However, in the case of antigens with abundant expression on tumor cells, standard-affinity antibodies should be adequate for killing leukemic cells while sparing normal cells with low levels of the antigen. In this context, high affinity could be counterproductive given the sequestration of such antibodies by antigen sinks in normal tissues or by affinity barriers.^113, 114^

Several bispecific antibody platforms have been tested in AML. Each platform has its unique strengths and weaknesses. BiTEs, diabodies and DARTs have small molecular size (~50 kD) below the renal clearance threshold. Although suitable for imaging because of their fast clearance from the blood, continuous infusion is necessary for their therapeutic applications. Other formats, such as Fc-fusion proteins or IgG-scFv have molecular sizes above the renal threshold and hence longer serum half-lives. None of the platforms presented in this review are able to cross the blood brain barrier, which permits only certain molecules with small molecular weights (⩽600 Da) and water–lipid partition coefficients, unless the bispecific antibody is piggybacked onto transcytosis pathways such as the transferrin receptor.^115^ In addition to their size, the valency of these platforms could be important for their biologic effect. Higher avidity for their cognate antigen usually translates into better binding and cytotoxicity, although cross-linking antigens can also lead to internalization of some antigens (for eample, CD33). Bispecific antibodies that engage T cells stimulate the release of proinflammatory cytokines which act like a double-edged sword. Such cytokines can increase the anti-leukemia efficacy by direct cytotoxicity and by activation and recruitment of immune cells into the tumor site. However, they also increase the risk of adverse effects including the life-threatening cytokine release syndrome.^116^ Moreover, lessons learned from bone marrow transplantation would suggest that application of bispecific antibodies at the time of minimal residual disease should maximize the chance of cure besides reducing the risk of tumor lysis syndrome or the accompanying cytokine storm.

Generally, antibodies recognize cell surface antigens. Nonetheless, as the majority of leukemia-associated antigens are intracellular proteins whose peptide epitopes are presented outwardly in the context of HLA molecules, developing bispecific antibodies against these peptide–HLA complexes can unveil a treasure trove of potential novel tumor-specific targets. However, the field of TCR-like antibody is still in its infancy and many challenges remain. Cross reactivity of such antibodies with other peptides and with other HLA molecules could risk adverse side effects. Furthermore, as these antibodies only recognize antigens in the context of HLA molecules, they can only be applied in patients with specific HLA types.

Although not covered in this review, highly encouraging results of chimeric antigen receptor (CAR)-armed T cells in lymphoblastic leukemia have been widely published. Yet the cost and complexity of cell preparation protocols, as well neurotoxicity could hamper the universal application of CAR technology beyond specialized centers with well-trained cytotherapy staff. In comparison, bispecific antibodies could be more cost-effectively generated in pharmaceutical scales for conventional transport and distribution with less geographic restrictions. Despite these advantages, the future of bispecific antibodies for AML will depend highly on their clinical efficacy and toxicity as more patients are being treated with these modalities.

## Figures and Tables

**Table 1:** Advantages and disadvantages of AML-associated antigens for antibody development (for a cartoon representation of each bispecific antibody format, please see Figure 1)

*Antigen*	*Advantages*	*Disadvantages*	*Bispecific formats*
CD33	Expressed on the majority of myeloid blasts and leukemic stem cells (LSC)^6, 117^ Not expressed or expressed at lower levels on normal HSCs^6, 117^ Not expressed outside of the hematopoietic system^118^ High CD33 expression is associated with high-risk mutations and inversely associated with low-risk mutations^6, 119^ High CD33 expression correlated with inferior disease features and outcome^119^ Expressed on immunosuppressive myeloid derived suppressor cells that are associated with extramedullary infiltration and detection of minimal residual disease^120^	Not expressed on all myeloid blasts or myeloid stem cells^117^ Expressed on HSCs in some studies^121^ Expressed on activated natural-killer (NK) cells and T cells^5, 9^ Modulation (decreased surface expression) of surface CD33 expression upon bivalent anti-CD33 antibody treatment^10, 122^ Circulating CD33 might interfere with the anti-CD33 antibodies^123^ Treatment-related myelosuppression (neutropenia and thrombocytopenia)^124^ Relatively low abundance on cell surface^125^	Tandem double and triple scFv (BiTE, ScBsTaFv, bsscFv, TandAb, BiKE, TriKE, sctb), chemical conjugates
CD123	Expressed on the majority of myeloid blasts and LSCs^126^ Not expressed or expressed at lower levels on normal HSCs^38, 121^ Absent on T cells^127^ Associated with lower complete remission and poorer survival rates^39^ Associates with higher proliferation and more resistance to apoptosis of AML cells^39^	Expressed on HSCs^121, 128^ Not expressed on all myeloid blasts or myeloid stem cells^39^ A chimeric antigen receptor that was made based on CD123 antibody significantly reduced B cells, platelets and myeloid cells in an animal model^128^	Tandem double and triple scFv (sctb), DART, BIf
WT1	Expressed on the majority of myeloid blasts and LSCs^44, 129^ Is not expressed or expressed at extremely low levels in a small population of CD34(+)cells in bone marrow^129^ Higher WT1 gene expression is associated with lower complete remission and decreased survival^45, 130^	HLA-restricted expression of WT1 limits the application of each anti-WT1 antibody to one special HLA haplotype^46^ Not expressed on all myeloid blasts or myeloid stem cells^129^ Low cell surface density of HLA-WT1-peptide complexes^46^ Expressed in some normal tissues^131^	Tandem double scFv (BiTE)
CD13	Expressed on the majority of myeloid blasts and LSCs^132^ Expressed at higher levels on AML stem cells than on normal HSCs Anti-CD13 monoclonal antibodies can induce apoptosis in AML cells	Expressed in some normal tissues and cells including monocytes, granulocytes, capillary endothelium, nephron convoluted tubules, bile ducts, pancreas, skin, small intestine and liver^133^ Not expressed on all myeloid blasts^132^ Expressed on HSCs^121^	Chemical conjugation (Fab′ fragments)
CD15	Expressed on the majority of AML cells^134^	Expressed on some NK cells, T cells, monocytes, neutrophils, eosinophils and neurons^52, 135^ Expressed on more than 50% of activated T cells^136^	Chemical conjugation (Fab conjugated with whole IgM)
CD30	Associated with FLT-3-ITD mutations, leucocytosis and possibly poorer prognosis^57^	Expressed on respiratory epithelial cells, glandular cells of gallbladder, colon, rectum and uterus^137, 138^ Expressed on activated T cells^55^ Not expressed on all AML cells^56, 57^ Secreted upon cleavage of the extracellular domain^58, 59^	T and Ab
CD45	Not internalized upon antibody ligation^139^ Expressed on most AML cells^140^ Not expressed outside hematopoietic system	Not expressed on LSCs^141^ Expressed on all hematopoietic cells except mature red blood cells and platelets^61^	
CD47	Is a universal target in human cancers CD47 upregulation on leukemic cells allows them to evade macrophage killing^64^ Overexpressed on AML stem cells than on their normal hematopoietic counterparts^65^ Overexpression of CD47 on AML cells is associated with shortened survival^65^	Expressed on the majority of normal tissues^63^ CD47 expression on normal tissues may generate an antigen sink preventing the therapeutic antibody to reach its target on AML cells^66^	DVD-Ig
CLL1	Expressed on the majority of myeloid blasts and LSCs^142^ Not expressed on normal tissues^142^ Not expressed on normal hematopoietic stem cells^67, 142^	Expressed on peripheral blood monocytes, dendritic cells and granulocytes^142^ Relatively low abundance on cell surface^142^ Antigen modulation upon ligation with anti CLL1 antibody^142^ Not expressed on all AML cells^142^	Chemical conjugation (Fab fragments)
FLT-3	Expressed on the majority of AML samples^143^ Expression on LSCs is higher than on normal hematopoietic cells^70, 73^ Expressed on LSCs^143^	Expressed on hematopoietic stem and progenitor cells and on dendritic cells^70, 71^ Not expressed on all AML cells^143^	Tandem double scFv (BiTE), Fabsc
VEGF-A, Ang-2	VEGF-A and Ang-2 are overexpressed on the majority of AML bone marrow samples^144^ Anti-angiogenic therapy might control disease in patients with relapsed AML^144^ Expression of VEGF-A and Ang-2 on AML is associated with negative outcome^74, 75, 144^	Not expressed on all AML cells^144^ Are secreted from the cells so T-cell responses cannot be redirected against AML cells	CrossMab, chemical conjugation

**Table 3:** Characteristics of bispecific antibodies generated for AML

	*MW (kDa)*	*Affinity(*K_*D*_*) nm*			*EC*_*50*_*pm*	*Clone*
		*Effector (CD3 and so on)*	*Target (CD33 and so on)*			
			*Parent clone*	*Bispecific format*		
CD33 × CD3 BiTE AMG 330^(ref.^^9)^	55	5.1	—	8	0.4–3	—
CD33 × CD3 scBsTaFv^17, 146, 147^	60	—	0.1	10–100	15	DRB2 (CD33)
CD33 × CD16 chemically conjugated^23^	—	—	—	—	—	251 (CD33), 3G8 (CD16)
CD16 × 33 BiKE^148, 149, 150^	55	20 (scFv)	—	—	—	NM3E2 (CD16)
CD33 × CD16 × CD33 sctb^29^	90	45.1±4.3	—	7.9±1.1	1.8–18	K132 (CD33), 3G8 (CD16)
CD33 × CD16 × CD19 sctb^30^	90	49±5.2	—	CD33 (29±1.9)	7.2±2	K132 (CD33), 3G8 (CD16)
CD123 × CD16 × CD33 sctb^31^	90	21.7±1.8	—	CD33 (17.8±2.2), CD123 (18.8±0.9)	21–118	K132 (CD33), 3G8 (CD16), CD123 (from phage^41^)
CD123 × CD3 BIF^151, 152^	140	10	0.01–0.05	0.1	10	12F1 (CD123), UCHT1 (CD3)
CD3 × CD123 DART^42^	59	9±2.3	—	0.13±0.01	0.17	7G3 (CD123)
CD123 × CD16 bsscFv^41^	60	49±5	—	4.5–101	211–364	3G8 (CD16), CD123 (from phage^41^)
CD15 × CD64 chemically conjugated^54^	—	—	—	—	—	PM81 (CD15), 32 (CD64)
CD33 × CD64 chemically conjugated^32, 34^	—	—	—	—	—	H22 (CD64), 251 (CD33)
CD16 × CD33 chemically conjugated^23^	—	—	—	—	—	3G8 (CD16), 251 (CD33)
CD3 × CD33 modular re-targeting system^19^	—	—	0.1	—	—	MT-301 (CD3), DRB2 (CD33)
CD33 × CD3 BiFab^a,68^	~100	—	7	Similar to their parental clones	25–445	Mutated hM195 (CD33), and UCHT1 (CD3)
anti-CD3 Fab′ × anti-CD13 Fab′ chemically conjugated^51^	100–110	—	—	—	—	OKT3 (CD3), My7 (CD13)
CD30 × CD16A TandAb^60^	105–110	0.39	17.2	9.3	35 800	LSIV21 (CD16), HRS-3 (CD30)
CD30 × CD16A bispecific diabody^60^	60	35	17.2	751	194 700	LSIV21 (CD16), HRS-3 (CD30)
WT1 × CD3 BiTE^46, 47^	—	—	—	0.2	—	ESK1 (WT1), L2K (CD3)
CD20 × CD47 DVD-Ig	—	—	CD47 (3.1)	CD47^(refs^^48–60)^	—	2B8 (CD20), B6H12.2 (CD47)
CLL1 × CD3 BiFab^a,68^	~100	—	6.1	Similar to their parental clones	2.1–41	Mutated 1075.7 (CLL1), and UCHT1 (CD3)
FLT-3 × CD3 BiTE^71^	—	—	—	—	—	4G8 (FLT-3),UCHT1 (CD3)
FLT-3 × CD3 Fabsc^71^	87	—	—	—	—	4G8 (FLT-3), BV10 (FLT-3), UCHT1 (CD3), OKT3 (CD3), BMA031 (CD3)
VEGF × Ang-2 CrossMab^80, 81^	—	—	Bevacizumab (<0.1) Ang-2 (0.2)	Bevacizumab (<0.1) Ang-2 (0.2)	—	Bevacizumab=humanized A.4.6.1 (VEGF) and LC06 (Ang-2)

a BiFab antibodies were generated by conjugating the antigen-binding fragments (Fab) of two antibodies using bio-orthogonal chemical linkers.

**Table 2:** Clinical trials involving bispecific antibodies in AML

*Name*	*Format*	*Phase*	*Endpoint*	*Clinical trial number*	*Sponsor*	*Study population*	*Status*
JNJ-63709178	CD123 × CD3 DuoBody	1	Safety and efficacy	NCT02715011	Janssen Research & Development, LLC	Relapsed/refractory AML	Suspended
AMG 330	CD33 × CD3 Tandem scFv (BiTE)	1	Safety	NCT02520427	Amgen	Relapsed/refractory AML	Suspended
MGD006	CD123xCD3 DART	1	Safety	NCT02152956	MacroGenics	Relapsed/refractory AML, MDS	Recruiting

Abbreviations: AML, acute myeloid leukemia; BiTE, bispecific T-cell engager; DART, dual-affinity re-targeting; MDS, myelodysplastic syndrome.

DuoBody (Genmab, Copenhagen, Denmark) is the commercialized name of the controlled Fab-arm exchange (cFAE) platform to generate IgG1 monovalent bispecific antibodies.^145^
